# Detection of Hemoglobin Constant Spring: A Comparison of Capillary Electrophoresis Versus High-Performance Liquid Chromatography

**DOI:** 10.7759/cureus.67228

**Published:** 2024-08-19

**Authors:** Sharifatul Fatma Embong, Adibah Daud, Mohammad Hudzaifah Nordin, Sumaiyah Adzahar

**Affiliations:** 1 Faculty of Medicine, Universiti Sultan Zainal Abidin, Kuala Terengganu, MYS; 2 Department of Pathology and Medical Laboratory, Faculty of Medicine, Universiti Sultan Zainal Abidin, Kuala Terengganu, MYS

**Keywords:** high-performance liquid chromatography (hplc), non-deletional α-thalassemia, alpha-thalassemia, capillary electrophoresis, hemoglobin constant spring

## Abstract

Introduction: In Malaysia, Hemoglobin Constant Spring (Hb CS) is the most common non-deletional α-thalassemia, caused by a mutation at the termination codon of the α2-globin gene (TAA>CAA). Detection typically involves identifying an abnormal peak at zone 2 on capillary electrophoresis (CE) or a small peak at the C-window on high-performance liquid chromatography (HPLC), indicative of Hb CS.

Objective: This study aimed to investigate the correlation between HPLC and CE in detecting Hb CS, evaluating their respective diagnostic accuracies and limitations.

Methods: A cross-sectional study was conducted at Hospital Sultanah Nur Zahirah involving secondary school students (Form 4) from Terengganu who participated in a thalassemia screening program conducted by the Ministry of Health (MOH) from January 2019 to December 2022. Blood samples from subjects showing a positive peak in zone 2 of CE and a small peak at the C-window of HPLC were selected. Molecular studies of these samples were performed to confirm the presence of Hb CS. For the statistical analysis, the Pearson correlation coefficient was employed to assess the relationship between CE and HPLC results.

Results: Hb CS was confirmed in all samples by molecular studies, revealing 92.3% heterozygous, 7.2% compound heterozygous, and 0.5% homozygous cases. CE detected 92.3% of heterozygous Hb CS cases, while HPLC detected only 48.2%. For compound heterozygous Hb CS, CE detected 100%, whereas HPLC detected 89.3%. Both homozygous cases were detected by CE and HPLC. The Pearson correlation coefficient test showed a significant linear relationship (p<0.001) between CE's zone 2 peak values and HPLC's C-window peaks (n=389).

Conclusion: These findings highlight the efficacy of CE as a reliable method for Hb CS detection, suggesting its potential superiority over traditional HPLC in clinical settings.

## Introduction

Thalassemia is a group of inherited blood disorders characterized by abnormalities in α- and β-globin synthesis, leading to ineffective erythropoiesis and hemolysis, resulting in anemia and various complications [1]. There are two main types of thalassemia, alpha and beta, both inherited similarly and diagnosed through hematologic tests and DNA analysis [2]. α-Thalassemia is a hereditary blood disorder caused by mutations in the α-globin gene, leading to a deficiency in α-globin chains and affecting hemoglobin production [3]. It presents a spectrum of clinical manifestations, from mild anemia to severe conditions like hydrops fetalis. The condition is prevalent in regions like southern China, Middle Eastern and Mediterranean countries, Southeast and South Asia, and Africa and around the Mediterranean [4]. The condition can be broadly classified into two types based on the genetic mutations involved: deletional and non-deletional α-thalassemia [5].

Hemoglobin Constant Spring (Hb CS) is the most common non-deletional α-thalassemia in Southeast Asia, with a gene frequency of 3-6% [6]. It is caused by a point mutation at the termination codon of the HBA2 gene, resulting in 31 additional amino acids, and due to the abnormal structure and unstable mRNA, the rate of αCS-globin chain synthesis is markedly decreased. Hb CS can exist in different genotypes, including heterozygous, compound heterozygous, and homozygous forms, each with varying levels of Hb CS aiding in their differentiation [7]. The Hb CS heterozygote is clinically and hematologically normal. However, the homozygote presents with symptoms of α- or β-thalassemia intermedia, including mild anemia, jaundice, and hepatosplenomegaly [8]. Additionally, the co-inheritance of Hb CS with various determinants of α-thalassemia produces a broad spectrum of clinical and hematological phenotypes, ranging from normal to intermediate thalassemia. For example, the co-inheritance of Hb CS with a single α-3.7 deletion results in a clinical phenotype similar to two α-gene deletions. Conversely, the co-inheritance of Hb CS and Hb Adana, another mutational type, can result in more severe anemia [9,10].

The Hb CS mRNA is unstable, resulting in a decreased rate of α-globin chain synthesis [11]. As a result, Hb CS is often missed by routine laboratory testing, especially in heterozygotes [9]. The conventional method for detecting thalassemia and abnormal hemoglobins, such as gel electrophoresis, may not distinguish and quantify the minute band of Hb CS. This can lead to misdiagnosis and improper treatment. There are three primary methods for detecting Hb CS [12]. Isoelectric focusing (IEF) electrophoresis is a manual technique that has been known to misdiagnose Hb CS diseases. Additionally, automated high-performance liquid chromatography (HPLC) can detect Hb CS in only 26-86% of Hb CS traits without providing accurate quantification. In contrast, capillary electrophoresis (CE) is an automated method with the highest reported sensitivity for Hb CS, ranging from 81% to 100% [6].

CE and HPLC are two widely used techniques nowadays for separating and identifying hemoglobin variants. This study focuses on the comparison between CE and HPLC in detecting Hb CS and explores their respective advantages and limitations.

## Materials and methods

Study subjects

This cross-sectional study utilized secondary data from the thalassemia registry and the hematology laboratory information system at Hospital Sultanah Nur Zahirah (HSNZ). The participants were secondary school students (Form 4) from Terengganu who took part in a thalassemia screening program conducted by the Ministry of Health (MOH) over a period of four years, from January 2019 to December 2022. The inclusion criteria were samples that showed a positive peak in zone 2 in CE within the study period. Samples collected post-transfusion within the last three months were excluded. Their blood samples were collected in EDTA containers and sent for hemoglobin analysis at HSNZ. A total of 10,630 samples were analyzed during this study period, and 584 samples exhibited a peak in zone 2 CE. However, due to budget constraints, only 389 samples were randomly selected for DNA analysis. Ethical approval was obtained from the National Medical Research Registry (NMRR) (approval number: ID-23-00137-ACP) and the Universiti Sultan Zainal Abidin Human Research Ethics Committee (UHREC) (approval number: UniSZA/UHREC/2023/569) and was carried out according to the Declaration of Helsinki.

Hemoglobin analysis

Samples were analyzed within 24 hours, first with a CE system (CAPILLARYS 2 Flex-Piercing System, Serbia) and then with HPLC (VARIANT II, Bio-Rad Laboratories, Hercules, California, United States) following the manufacturer's instructions. CE separates molecules based on their charge-to-mass ratio under the influence of an electric field within a capillary tube filled with an electrolyte. Hemoglobins, being proteins with different charges, migrate at different rates, allowing for their separation and identification. Hemoglobin was directly detected by measuring absorbance at 415 nm at the cathodic end of the capillary. The resulting electropherograms were analyzed for pattern abnormalities and the quantification of individual hemoglobin fractions. Up to 15 distinct zones can be identified on the electropherogram. In this study, the primary focus was the detection of Hb CS, which is found in zone 2. If a peak in zone 2 was observed on the CE electropherogram, a supplementary method, HPLC, was used to examine the peak in the C-window. HPLC separates components of a mixture based on their differing interactions with the stationary phase (column) and the mobile phase (solvent). Hemoglobin variants are separated as they pass through the column and are detected based on their retention times. The retention time or time of elution of any normal or variant hemoglobin detected is compared to known hemoglobins (A, F, and A2) and many other variants. Quantification is performed by integrating the area under each peak.

Molecular studies

Samples with a zone 2 peak in CE were sent for DNA analysis to the reference laboratory to confirm the presence of Hb CS. In Malaysia, the molecular analysis for α-thalassemia was conducted at Hospital Kuala Lumpur (HKL). The preferred method for detecting non-deletional α-thalassemia is the multiplex amplification-refractory mutation system (ARMS) PCR, which is based on the principle that a DNA polymerase can extend a primer only if it is perfectly matched to the target DNA sequence at its 3' end. In a multiplex setup, multiple primers are used in a single PCR reaction to simultaneously detect several mutations common in Malaysia, including the termination codon mutation TAA→CAA (Hb CS), codon 125 mutation CTG→CCG (Hb Quang Sze), codon 59 mutation GGC→GAC (Hb Adana), initiation codon mutation ATG→A‒G, codon 30 mutation ∆GAC, and codon 35 mutation TCC→CCC (Hb Évora), all within a single multiplex ARMS-PCR reaction.

Statistical analysis

Data analysis was conducted with IBM SPSS Statistics for Windows, Version 26.0 (Released 2019; IBM Corp., Armonk, New York, United States). The Pearson correlation coefficient was used to determine the relationship between CE and HPLC results. Statistical significance was set at p<0.05.

## Results

A total of 389 samples were analyzed, consisting of 140 (36%) male and 249 (64%) female. The study was predominantly Malay population because it was conducted in Terengganu, with 388 (99.7%) participants being Malay and one (0.3%) being Chinese. Demographic data of the participants are shown in Table 1. 

**Table 1:** Demographic data of the participants (n=389)

Variables	n (%)
Gender	
Male	140 (36)
Female	249 (64)
Race	
Malay	388 (99.7)
Chinese	1 (0.3)

Distribution and genotypes of Hb CS

Molecular studies confirmed Hb CS in 389 samples. Among these, 359 samples (92.3%) were heterozygous Hb CS, 28 samples (7.2%) were compound heterozygous Hb CS, and two samples (0.5%) were homozygous Hb CS (Table 2).

**Table 2:** Genotypes of Hb CS (n=389) Hb CS: Hemoglobin Constant Spring

Genotypes of Hb CS	n (%)
Heterozygous Hb CS	359 (92.3)
Homozygous Hb CS	2 (0.5)
Compound heterozygous Hb CS	28 (7.2)

Detection of Hb CS by CE and HPLC

Of the heterozygous Hb CS cases, 359 (92.3%) were detected by CE, whereas only 173 (48.2%) were detected by HPLC. For compound heterozygous Hb CS, CE detected 28 (100%) cases, whereas HPLC detected 25 (89.3%) cases. Both homozygous cases can be detected by CE and HPLC (Table 3). 

**Table 3:** Detection of Hb CS by CE and HPLC samples (n=389) Hb CS: Hemoglobin Constant Spring; CE: capillary electrophoresis; HPLC: high-performance liquid chromatography

Genotypes of Hb CS	CE	HPLC	
	Zone 2 n (%)	No peak n (%)	Small peak n (%)
Heterozygous Hb CS	359 (92.3)	186 (51.8)	173 (48.2)
Homozygous Hb CS	2 (0.51)	0 (0)	2 (100)
Compound heterozygous Hb CS	28 (7.2)	3 (10.7)	25 (89.3)

**Figure 1:**
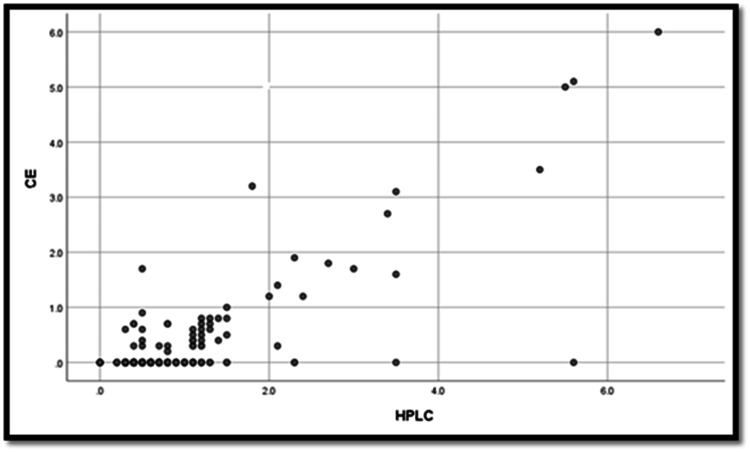
Scattered plot showing the correlation between CE and HPLC CE: capillary electrophoresis; HPLC: high-performance liquid chromatography

The Pearson correlation coefficient test was used to examine the relationship between HPLC and CE findings of Hb CS by comparing the peak value in zone 2 of CE with the small peak detected on HPLC at the C-window (n=389). A significant linear correlation was found between the HPLC and CE values (p<0.001) (Figure 1), with a Pearson correlation coefficient (r) of 0.663, indicating a moderate to good correlation.

## Discussion

Hb CS is an elongated thalassemic α-chain variant commonly found in Southeast Asian populations. Due to its small quantity and instability, it is often misdiagnosed by standard methods such as gel electrophoresis and HPLC [13]. In this study, the efficacy for detecting and quantifying Hb CS was compared between the CE and HLPC methods in the three types of Hb CS, including heterozygous, homozygous, and compound heterozygous forms.

In the HPLC analysis, the Hb CS chromatogram exhibited at least a small peak in the C-window with a retention time of 4.90-5.30 minutes. This peak was observed in two (100%) of subjects with homozygous Hb CS, 25 (89.3%) with compound heterozygous Hb CS, and 173 (48.2%) with heterozygous Hb CS. When analyzed by CE, 359 out of 389 samples (92.3%) showed a positive peak in zone 2 and were identified as heterozygous Hb CS. The remaining samples, two (0.51%) and 28 (0.72%), were detected as homozygous Hb CS and compound heterozygous Hb CS, respectively. In this study, CE correctly identified all Hb CS cases, demonstrating an advantage over HPLC for detecting the Hb CS variant. CE is suitable for the identification and quantification of both common and rare Hb variants, producing results that are comparable with existing HPLC and electrophoretic methods [14].

This study demonstrated a moderate to good correlation between HPLC and CE findings for Hb CS detection, as indicated by the Pearson correlation coefficient test. CE patterns were found to be easier to interpret than HPLC patterns for Hb CS, showing a distinct peak in zone 2 of CE compared to a small bump in the C-window of HPLC. Additionally, most samples detected by HPLC lacked quantitative measurement. CE is a powerful analytical technique used to separate ionic species based on their size-to-charge ratio. It has been widely adopted for hemoglobin variant analysis due to its high resolution, speed, and automation capabilities [15]. The comparison between CE and HPLC in the detection of Hb CS has been the subject of several studies [16]. The consensus in the literature suggests a high degree of correlation between the two methods, although each has unique advantages [17]. One study by Waneesorn et al. [18] compared CE and HPLC for the detection and quantification of Hb CS. The study demonstrated a strong correlation between the two methods, with correlation coefficients often exceeding 0.95, indicating high agreement in quantifying Hb CS levels. This supports the reliability of both techniques for clinical diagnostics. Another study by Pornprasert and Punyamung [19] evaluated the use of CE for quantifying Hb CS in both heterozygous and homozygous individuals. The findings confirmed that CE is effective in quantifying Hb CS, and the results were consistent with those obtained using HPLC, further validating the use of CE alongside HPLC in clinical settings. While both techniques demonstrated high sensitivity and specificity for Hb CS detection, CE was noted for its faster analysis time, whereas HPLC offered slightly better reproducibility. In clinical settings, the choice between CE and HPLC may depend on specific needs. According to Çakır Madenci et al. [20], CE is preferred for rapid screening due to its speed and ease of use, while HPLC is often used for confirmatory testing owing to its detailed quantification capabilities.

A limitation of this study was that, out of 584 samples showing a peak value in zone 2 of CE, not all were subjected to molecular DNA confirmation tests due to financial constraints. Consequently, the total number of samples included in the study was reduced. To address this issue in the future, a collaborative study with other institutions could be considered, as it might help reduce costs and increase the number of samples processed.

## Conclusions

While both CE and HPLC are valuable tools for detecting Hb CS, CE offers higher sensitivity and easier interpretation, making it particularly advantageous for identifying and quantifying Hb CS. However, HPLC remains widely used due to its ability to detect multiple hemoglobin variants and established protocols. The choice between these methods will depend on the specific requirements of the laboratory, including cost, available equipment, and the need for sensitivity versus versatility.
